# Identifying Developmental Motor Difficulties: A Review of Tests to Assess Motor Coordination in Children

**DOI:** 10.3390/jfmk5010016

**Published:** 2020-02-24

**Authors:** Alice Cancer, Rebecca Minoliti, Maura Crepaldi, Alessandro Antonietti

**Affiliations:** 1 Department of Psychology, Catholic University of the Sacred Heart, 20123 Milan, Italy; rebecca.minoliti01@icatt.it (R.M.); alessandro.antoniett@unicatt.it (A.A.); 2 Department of Human Science, University of Bergamo, 24129 Bergamo, Italy; m.crepaldi@studenti.unibg.it

**Keywords:** developmental coordination disorder, dyspraxia, assessment, developmental neuropsychology

## Abstract

The latest guidelines recommend early identification of children with motor impairments using a standardized norm-referenced test. Motor coordination difficulties in developmental age have been studied extensively over recent years, with experimental literature on developmental coordination disorder (DCD) suggesting that motor proficiency assessments depend on the nature of the task at hand. In this article we reviewed 14 assessment tools to measure movement performance in childhood and adolescence, which are often referred to in an international context. This updated review aims to compare motor tests depending on a) the nature of the tasks included in the battery (i.e., questionnaire and clinical examination), b) psychometric properties, and c) cultural adaptation to relevant developmental norms. Finally, implications for diagnosis and clinical practice are discussed. Considering there are several tests used for DCD, it is important to better define their reliability and validity in different cultures in order to better compare the validation studies and select the most appropriate test to use in the assessment procedure.

## 1. Introduction

The term developmental coordination disorder (DCD; DSM-5: 315.4) [1], or specific developmental disorder of motor functions (SDDMF; ICD-10: F82,) [2], refers to children with developmental motor difficulties [3]. In the previous DSM edition (DSM-IV-TR), DCD was included under the category ‘learning disorders’, however in DSM-5 it is categorized as a motor disorder within the comprehensive category of ‘neurodevelopmental disorders’. According to Criterion A of DSM-5 requirements for diagnosis, acquisition and execution of coordinated motor skills are below those expected for age and learning opportunities. DCD-related difficulties are manifested as clumsiness and as slowness as well as inaccuracy of performance of motor skills. Furthermore, DCD children show impairments in praxic functions, such as planning and/or recovering actions, producing voluntary daily life gestures which involve the use of objects (e.g., brushing teeth, catching objects, using scissors or cutlery, handwriting, riding a bike, or participating in sports), assuming body postures with no-sense (e.g., grasping the right wrist with the left hand and vice versa) or postures with a functional signification (e.g., the sign of victory). Diagnoses of DCD cannot be based on the Criterion A alone. Motor coordination difficulties also have to significantly or persistently interfere with academic achievement or activities of daily living (Criterion B). Moreover, an additional criterion is included in DSM-5: The onset of symptoms occurs during the developmental period [4]. DCD has a prevalence of 5–6% in the infant population [3]. The scientific literature indicates that one of the most frequent risk factors for the development of DCD is preterm birth [5] and perinatal issues (e.g., intrauterine growth retardation, low Apgar index, cerebral palsy) [6,7]. However, to date the neuroanatomical and functional etiology of the DCD in children born at term has not yet been clarified [8,9], and no consensus has been established about the etiology of DCD [10,11,12,13]. For the diagnosis of DCD, the motor skill deficits are not better explained by other motor or intellectual disabilities or visual impairment, and are not linked with a neurological condition (e.g., cerebral palsy, muscular dystrophy, degenerative disorder) [10]. The diagnosis of DCD requires a systematic evaluation, with an adequate anamnesis, specific tests, and a multidisciplinary comparison between different professionals that together collaborate to develop a functional profile of the subject for the purposes of both the diagnosis and a targeted rehabilitation project [3]. Intragroup approach through factor and cluster analysis highlights that motor impairment in DCD children varies both in severity and nature. Accordingly, studies have used screening measures of developmental milestones performance derived from global motor tests. Although there is a strong body of work that assessed combinations of different factors involved in DCD [14,15,16,17], few studies have assessed both motor and cognitive abilities using a standardized test, such as neuromuscular tone and soft signs evaluation, qualitative and quantitative measures of gross and fine motor coordination, along with specific academic, language, visual–perceptual/visual–motor, and attentional/executive difficulties. This approach would allow a better identification of DCD subtypes by providing an understanding of the mechanisms and of the cerebral involvement [10]. The aim of the present review is the comparison of the most used motor tests depending on (a) the nature of the tasks included in the battery (i.e., questionnaire and clinical examination), (b) psychometric properties, and (c) cultural adaptation to relevant developmental norms.

## 2. Motor Coordination Assessment Tools

To date, several tools are used internationally in the healthcare practice for identifying and assessing motor coordination disorders. These tools can be divided into (a) standardized motor performance tests, administered by qualified professionals (see the mentioned Criterion A), and (b) self-report questionnaires and observational instruments developed for parents, teachers, and/or healthcare professionals which are useful for evaluating the impact of coordination difficulties on children’s daily life motor activities (Criterion B). Earlier reviews of motor coordination tests in the developmental age focused on specific age ranges, such as preschoolers [18,19] or young adults [20]. A search of the literature, using a snowballing approach (search terms: ‘motor’, ‘developmental coordination disorder’, ‘assessment’, ‘test’; electronic databases: PsycInfo and PubMed) was made to provide an updated and comprehensive review of assessment tools for identifying motor coordination disorders in the developmental age (see Table 1).

### 2.1. Motor Performance Measures

#### 2.1.1. The Motoriktest für vier-bis sechsjährige Kinder (MOT 4–6)

The Motoriktest für vier-bis sechsjährige Kinder (The Motor Test for four- to six-year-old Children; MOT 4–6) is a German test developed by Zimmer and Volkamer in 1987 [21]. It allows an assessment of the most important fine and gross motor skills, and identification of early problems or deficits in movement development. The test is composed of 18 items, analyzing locomotion, stability, object control, and fine movement skills. The quantitative point attribution rates on a 3-point scale from 0 (skill not mastered) to 2 (skill mastered). In addition, a qualitative comment about the child performance is provided. The scale has an adequate internal consistency (α = 0.81). No translation of its manual is available yet, therefore the MOT 4–6 has been used in studies conducted mainly in German-speaking countries.

#### 2.1.2. The McCarron Assessment of Neuromuscular Development (MAND)

The McCarron Assessment of Neuromuscular Development (MAND) [22] was originally designed by McCarron in 1982 as a screening and assessment tool to be used by health professionals with children aged 3.5–16 years old. MAND measures fine and gross motor abilities, identifies children with potential developmental problems, and also describes changes in motor behaviors associated with physiological pathologies. The test comprises of 10 items, five fine motor, and five gross motor. Normative tables are provided at 6-monthly intervals from 3.5–12 years, 12-monthly intervals to 16 years, one for above 16 years. A great advantage of the test is that it contains quantitative and qualitative items. For example, a fine motor quantitative item considers the number of beads moved from one box to another in 30 s, repeated for both hands; A gross motor qualitative item estimates how the subject walks forwards and backward along a 10 ft line.

#### 2.1.3. The Peabody Developmental Motor Scales–2nd Edition (PDMS-2)

The Peabody Developmental Motor Scales–2nd Edition (PDMS-2) was published in 2000 by Folio and Fewell [23]. The original version was created by the same authors in 1983 [38]. The PDMS-2 measures fine and gross motor skills. The scale allows the assessment of motor competence of children from birth to six years old relative to their peers. It comprises four subtests about gross motor skills and two subtests about fine motor skills. The four gross motor subtests cover reflexes (eight items), stationary performances (30 items), locomotion (89 items), and object manipulation (24 items). The two fine motor subtests cover grasping (26 items) and visual–motor integration (72 items). The total score is given by the sum of the points of each subscale. Each item has to be rated on a 3-point rating scale, of which 2 represents an attained skill, 1 a developing skill, and 0 a non-acquired skill. The internal consistency of the scale is very high (α = 0.97).

#### 2.1.4. The Test of Gross Motor Development—2nd Edition (TGMD-2)

The Test of Gross Motor Development—2nd Edition (TGMD-2) of 2000 [24] is the revision of the original Test of Gross Motor Development (TGMD) published by Ulrich and Sanford in 1985 [39]. The test measures gross movement skills and gives a qualitative evaluation in children from 3–10 years old. TGMD-2 helps to identify those children who are significantly behind their peers in gross motor development and, after that, to plan improvement interventions. The test includes the assessment of 1) locomotion abilities (e.g., running, galloping, hopping, leaping, horizontal jumping and sliding) and 2) objects control skills (e.g., two-hand striking a stationary ball, stationary dribbling, catching, kicking, overhand throwing and underhand rolling). The child has to perform each item twice. One point is given when the performance is correct, whereas 0 when it is wrong. The total score is the sum of every item repeated twice. The internal consistency coefficients go from α = 0.85 to α = 0.91.

#### 2.1.5. The Maastrichtse Motoriek Test (MMT)

The Maastrichtse Motoriek Test (Maastricht Motor Test; MMT), by Vles, Kroes, and Feron [26], combines a qualitative evaluation (36 items) with a quantitative measurement (34 items) of movement skill performance. MMT defines fine as well as gross motor capabilities of 5–6 years old children. MMT is composed of a total of 70 items. The score for each item varies from 0 to 2 points. The test must be administered by trained professionals, especially for the score of qualitative aspects of movement, which requires well-trained observation skills. The advantage of the MMT is that it gives a holistic view of the child’s strengths and weaknesses. The disadvantages are the absence of locomotor skill items and the narrow age range covered.

#### 2.1.6. The Bruininks-Oseretsky Test of Motor Proficiency (BOTMP)

The Bruininks-Oseretsky Test of Motor Proficiency (BOTMP) was firstly designed in 1978 [40] and it was later revised by Bruininks and Bruininks [27]. It allows the assessment of fine manual control, manual coordination, body coordination, strength, and agility, and it is useful to identify motor skill problems and/or specific motor deficits. It provides a complete form with 53 items and a short form with 14 items. BOTMP is composed of eight subtests (i.e., fine motor precision, fine motor integration, manual dexterity, bilateral coordination, balance, running speed and agility, upper-limb coordination, strength) assessing four motor areas: (1) fine manual control, (2) manual coordination, (3) body coordination, and (4) strength and agility. Three composite scores can be calculated (i.e., total motor composite, fine motor composite, and gross motor composite). The test can be administered by professionals such as occupational therapists, special education professionals, physical therapists, and developmental adaptive physical education teachers. The short form takes 15–20 min to complete, whereas the complete form takes 45–60 min.

#### 2.1.7. The Körperkoordinationtest für Kinder (KTK)

The Körperkoordinationtest für Kinder (Body Coordination Test for Children; KTK) was first published by Kiphard and Shilling in 1974 (called “Hamm–Manburger Körperkoordination Test für Kinder”) [41] and it was revised by the same authors in 2007 [28]. The test estimates the general dynamic balance capabilities in children aged 5–14 with typical development and in children with brain damage, behavioral problems, or learning difficulties. That is, the only gross motor function evaluated by KTK is balance. The updated test is shorter than the first version (from six to four items) with the following skills being tested in the updated test; (1) walking backward along a balance beam, (2) moving sideways on boxes, (3) hopping for height on one foot, and (4) jumping sideways. This allows both a rapid administration and screening procedure. The KTK was used for the criterion validity studies of other assessment tools, e.g., the Movement Assessment Battery for Children (M-ABC 2) [29].

#### 2.1.8. The Movement Assessment Battery for Children—2nd Edition (M-ABC 2)

The Movement Assessment Battery for Children (M-ABC) was designed by Henderson and Sudgen in 1992 [42] and was later revised by Henderson, Sudgen, and Barnett in 2007 (M-ABC 2) [29]. M-ABC 2 is the best known and most widely used test for identifying and describing the movement difficulties of children and adolescents from 3.0–16.11 years old. The test provides an indication of children’s and adolescents’ motor functioning across fine and gross motor tasks. It is a standardized test comprising 24 subtests divided into eight activities the child is required to perform. Activities are divided into three components: manual dexterity, aiming and catching, and balance. M-ABC 2 permits the integration of quantitative and qualitative information in order to draw a comprehensive picture of the child’s motor performance. An observative checklist is also included for children aged 5–11 to be completed by teachers. The use of the M-ABC 2 battery is suggested by the European Guidelines on Coordination Development Disorder as part of the evaluation process in cases of suspected DCD [3]. M-ABC 2 is largely used around the world, having been translated in 10 different languages (e.g., Chinese, Dutch, Danish, Swedish, Italian, and Japanese) [43,44,45,46]. As for the psychometric properties of the test, the English manual does not report all indicators of validity and reliabilities; however, several peer-reviewed papers reports results of studies assessing the reliability of different cultural adaptations of the battery (e.g., [34,47]).

#### 2.1.9. Abilità Prassiche e della Coordinazione Motoria—2nd Edition (APCM-2)

The test Abilità Prassiche e della Coordinazione Motoria (Praxic and Motor Coordination Skills)—2nd Edition (APCM-2) is an Italian test created and validated by Sabbadini [31]. It measures both motor and praxic coordination, to better characterize profiles of DCD or dyspraxia, from 24 months. The stated purpose of the test is the early identification of motor coordination disorder risk signs in toddlers, preschoolers, and children (2–8 years). The test includes six versions of the questionnaire for specific age groups: 24–36 months (for which both short and complete versions are available), 37–48 months, 49–60 months, 61–72 months, and 6.1–8 years. The short form for toddlers has been designed to optimize the screening procedure in healthcare and educational settings, such as premature birth hospital units and preschools, whereas the complete form is recommended for clinical use by physicians, neuropsychologists, and neuromotor therapists. The test includes two sub-scales: (1) motor schemes (assessing balance and coordination, oculo-motivity, sequencing, and hand and fingers movement), and (2) adaptive cognitive functions (assessing dynamic coordination, graphomotor skills, manual skills, symbolic gestures, and constructive praxis abilities). The standardization sample comprised 261 children aged from 3–8 years, of which 54% were boys and 46% were girls. Internal consistency has been calculated for each of the two scales (motor schemes and adaptive cognitive functions); for both scales, α is higher than 0.75.

#### 2.1.10. The Zurich Neuromotor Assessment 2 (ZNA-2)

The Zurich Neuromotor Assessment (ZNA) was created by Largo, Fischer, and Caflish in 2002 [48]. A revised version of the test (ZNA-2) was developed by Kakebeeke and colleagues in 2018 [33]. The aim is to assess neuromotor development in subjects from 3–18 years old. The peculiarity of the test is that motor proficiency is modeled as a continuous function of age, so to allow the comparison of the motor performance of a child with that of same-age peers. The test is made up of 11 items, measuring five components of motor proficiency: fine motor, pure motor, dynamic balance, static balance, and movement quality. The same tasks are used for all ages and the performance in younger children is adjusted for by reducing the number of repetitions for children between 3–6 years.

### 2.2. Self-Report Questionnaires

#### 2.2.1. The Motor Observation Questionnaire for Teachers (MOQ-T)

The Motor Observation Questionnaire for Teachers (MOQ-T) is an 18-item screening questionnaire developed by van Dellen, Vaessen, and Shoemaker [49] to help teachers identify 5–11 years old children at risk for DCD. First called the Groninger Motor Observation Scale, the MOQ-T questionnaire has been largely used in both research and clinical practice. The original version of the questionnaire [49] was later revised by Schoemaker [25]. The revised version includes 18 items regarding both fine and gross motor functioning, which are grouped into two factors: (1) general motor functioning (e.g., ‘The child has difficulty performing activities involving whole-body movements like getting dressed, catching a ball’; ‘The child easily loses its balance’) and (2) handwriting (e.g., ‘The child has problems with tasks requiring eye-hand coordination, like handicrafts, writing’). Each item is rated on a 4-point scale (1 = never true for my child; 4 = always true for my child). The scale has a high internal consistency (α = 0.95).

#### 2.2.2. The Movement Assessment Battery for Children—2nd Edition (M-ABC 2) Checklist

The M-ABC 2 battery includes, along with the motor performance tasks, a teacher questionnaire, namely the M-ABC 2 checklist [29]. The checklist has both a motor and a non-motor component, thus providing information on direct and indirect factors that might affect movement. The motor part of the checklist contains 30 items divided into two sections: section A, which measures movement in a static and⁄or predictable environment; and section B, which measures movement in a dynamic and⁄or unpredictable environment. For each item, teachers have to rate the motor competence of a child on a 4-point scale (0 = very well; 3 = not close). The scale has a high internal consistency (α = 0.94) [35] and has been adapted in many different languages (e.g., Chinese, Dutch, Danish, Swedish, Italian, and Japanese) [43,44,45,46].

#### 2.2.3. The Developmental Coordination Disorder Questionnaire (DCDQ)

The Developmental Coordination Disorder Questionnaire (DCDQ) is a 15-item scale designed by Wilson and colleagues [50] to screen for coordination disorders in children aged 5–15 years. The questionnaire, which is designed to be completed by parents, provides a standardized method for measuring motor coordination of children in daily activities. Parents are asked to compare their child’s motor performance to that of his/her peers using a 5-point scale. According to the authors, parents are the most reliable respondents to report developmental problems of the child. DCDQ takes about 10–15 min to be complete, therefore providing a fast screening of motor difficulties. A revised version of the questionnaire with stronger psychometric properties was later proposed by the authors (DCDQ ’07) [30]. The DCDQ ‘07 measures three distinct factors: (1) ‘Control during movement’, which includes items related to the motor control of the child while performing a motor task (e.g., ‘Your child hits an approaching ball or birdie with a bat or racquet accurately’); (2) items concerning ‘fine motor and writing’ (e.g., ‘Your child’s printing or writing or drawing in class is fast enough to keep up with the rest of the children in the class’); and (3) ‘general coordination’, which includes items about sports, clumsiness, fatigue and learning new motor tasks (e.g., ‘Your child is quick and competent in tidying up, putting on shoes, tying shoes, dressing, etc.’). The three-factor scores alone do not provide any indication of the presence or absence of DCD, but the questionnaire provides support for the identification of difficulties in motor skills exhibited by the child. The scale has a high internal consistency (α = 0.88) [30]. The DCDQ ’07 is largely used worldwide and many adapted versions are available in different languages: Polish [51], Taiwanese [36], Brazilian [37], Italian [52], Dutch [53], French Canadian [54], German [55], Japanese [56], and Spanish [57].

#### 2.2.4. The Early Years Movement Skills Checklist (EYMSC)

The Early Years Movement Skills Checklist (EYMSC) was designed by Chambers and Sudgen [32,58] to be used flexibly by teachers, parents, and other professionals involved with children with movement difficulties. The authors aimed at implementing an efficient, easy-to-use, and accurate tool to assess movement skills of preschoolers from 3–5 years of age. The checklist comprises of a list of 23 items designed to paint a picture of the child’s functioning patterns in familiar and representative environments, such as home and school. The EYMSC is divided into four sections: section 1, which measures self-help skills (e.g., ‘The child can put on a T-shirt without assistance and the child can fasten accessible coat buttons.’); section 2: desk skills (e.g., ‘The child can copy a circle and a cross from a completed example.’ and ‘The child use scissors to cut across a 4” strip of paper.’); section 3: general classroom skills (e.g., ‘The child can sit on the floor with legs crossed and back straight.’ and ‘The child can move around the classroom/school avoiding collision with stationary people/objects.’; and section 4: recreational/playground skills (e.g., ‘The child can ride a variety of moving vehicles (pedal car, tricycle).’ and ‘The child can walk on tiptoes for 4 steps.’). The test is based on a 4-point scale. For each item there are four alternative responses which describes how well the child deals with the task: well (1), just (2), almost (3), not close (4). The scores obtained in each section are then added and these four separate totals are summed to achieve an overall score. The scale has been translated into many languages (e.g., Dutch, French, German, Hebrew, Norwegian, and Portuguese) [59,60].

## 3. Discussion and Conclusions

The latest DCD guidelines [3], which provide clarity over definitions, diagnosis, and intervention for children with DCD, recommend early identification of children with motor impairments [3]. Routes to assessment are usually initiated by concerns from parents, teachers, or pediatricians about late walking or difficulties with writing [61]. One of the markers of a mature motor system is the ability to adapt movement to a specific action in order to be more efficient and accurate. Children with DCD have a reduced ability to modify and adjust their movement in goal-directed actions e.g., altering the action pattern during motor execution [62]. Several studies confirm that children with DCD can plan simple movements as well as peers without DCD can, and that the major difficulties emerge in tasks and actions with increased complexity that require advanced motor planning skills [9,63]. These motor coordination difficulties are otherwise rarely considered in clinical assessment for other developmental disorders or behavioral problems. The assessment of motor skills in the developmental age should, other than providing an objective measure of motor performance which can be used to diagnose DCD, be used to recognize motor problems in different neurodevelopmental disorders. Furthermore, an accurate motor assessment is crucial to plan an effective intervention program. Indeed, evidence shows that performance on activities of daily living can be improved by effective intervention approaches [3].

The assessment tools for motor difficulties which are described in the present review include items and tasks designed for specific age ranges. The majority of the tests were designed for children older than five years, whereas only two of the 14 tests were developed to measure motor difficulties in the first two years of development, i.e., the PDMS-2 [23] and the APCM-2 [31]. However, these two tests do not have any other cultural adaptation, therefore their use is limited to United Kingdom and Italy, and the results are not comparable. Finally, whereas test–retest reliability was measured for PDMS-2, it was not for APCM-2.

Furthermore, four performance measures can be used in children from 3 or 3.5 years, i.e., MAND, M-ABC 2, ZNA, and TGMD. All these tests report test–retest reliability, but none report concurrent validity. Furthermore, KTK and ZNA have not been validated for the specific diagnosis of DCD [64].

Although there are no defined diagnostic markers for DCD, the early symptoms of motor problems can be identified during tasks such as standing, walking, and throwing and catching a ball [61]. Therefore, the observation of the child in day-to-day functioning gives the opportunity to measure the impact of motor difficulties on daily living tasks. Self-report questionnaires are the best tools for that, also with the advantage of being quick, accurate and, in most cases, not requiring a trained professional to administer them. These tests are especially relevant in the situation in which the professional evaluating the child does not have access to a comprehensive assessment battery, which includes standardized motor tests. Furthermore, since the manifestation of DCD can differ with context, gathering information from various respondents, who can observe the child in his/her daily activity (e.g., parents and/or teachers), can help the healthcare professional to paint a complete picture of the child motor functioning.

As for their psychometric properties, the four self-report questionnaires here described have adequate validity and reliability indicators, with moderate to high correlations with validated motor performance batteries (concurrent validity). Many cross-cultural adaptations of the M-ABC 2 checklist in both Europe and Asia–Pacific countries [29,65] and the DCDQ ’07 [30] have been made, confirming their strong psychometric properties and their sensitivity to identify DCD. A diagnosis of DCD requires information of both fine and gross motor skills, which are measured using standardized tests of motor performance. Such tests require the child to perform specific tasks regarding balance/stability, reflexes, locomotion/walking, object control, grasping, visual-motor integration, jumping, etc.

For self-report questionnaires special attention should be paid to those who fill in the questionnaire. Individual differences of responders should be taken into account, along with the cultural variations in parental care and expectations of motor behavior development in diverse contexts [66].

Among the 10 performance tests included in the present review, the M-ABC 2 battery is the most widely used in both DCD healthcare and research practice, with approximately 10 cultural adaptations. The M-ABC 2 provides a comprehensive evaluation of fine and gross motor abilities, which can be evaluated on both a quantitative and qualitative level. The battery also includes a checklist, making it the most versatile tool for both identifying DCD and designing the appropriate intervention. A weakness of this battery is that limited information on reliability is reported in the manual, even if several studies by researches from diverse countries measured inter-rater and test–retest reliability [67]. Considering foreign norms to interpret the motor measures is not recommended, and proper adaptation of each instrument should be used. The importance of cultural adaptation of assessment tools has been claimed by Tripathi and colleagues [68], according to which it is not possible to develop culturally sensitive assessment tools across geographical regions and environments. It is necessary to measure the cultural sensitivity of the test. For example, Vanvuchelen and colleagues [69] pointed out that the use of the PDMS-2 with American normative data in Flemish children was not reliable enough to distinguish between children with motor disorders and typically developing children, with an overestimation of the 5 year old Flemish preschool child. The importance of considering not only age and cultural differences but also gender differences, is pointed out in an Australian study about psychometric properties of MAND [66].

Fortunately, as shown by the present updated review of motor assessment tests, DCD professionals around the world have the possibility to use a variety of tools, specific for evaluation setting, age, culture, the aim of the assessment, and level of training of the administrator. The cautious selection of the test to use is critical for an early and accurate diagnosis and, most importantly, for an effective intervention that would change the motor developmental pathway of the child with motor difficulties. For this reason, we suggest that future work in this field should be oriented to better define cultural criteria and specific cut-offs to accurately identify DCD and describe the functional profile of the child. Cross-cultural studies are fundamental to compare results and their psychometric properties. Motor development is influenced by biological and cultural aspects, and this link should not be ignored. Professionals should be familiar with the diverse tools available to identify DCD, knowing their limitations and strengths, as to base their selection on the specific aims and context of the assessment procedure.

## Figures and Tables

**Table 1:** Motor coordination tests for children (i.e., checklists and motor performance tests).

Test	Authors and Year	Country, Language	Type	Age Range	N. Items	Test–Retest Reliability	Inter-Rater Reliability (Cohen’s k)	Internal Consistency (Cronbach’s α)	Concurrent Validity (M-ABC 2, r)	Cultural Adaptations
MOT 4–6	Zimmer & Volkamer (1987) [21]	Germany, German	Performance measure	4–6 yrs	18	0.85	0.88	0.81	NR	-
MAND	McCarron (1997) [22]	US, English	Performance measure	3.5–16 yrs	10	0.99	NR	NR	NR	-
PDMS-2	Folio & Fawell (2000) [23]	US, English	Performance measure	0–6.11 yrs	249	0.89–0.96	0.96	0.97	NR	-
TGMD-2	Ulrich (2000) [24]	US, English	Performance measure	3–10 yrs	12	0.91	0.84–0.96	0.85–0.91	NR	-
MOQ-T	Schoemaker et al. (2003) [25]	The Netherlands, Dutch	Self-report questionnaire	5–11 yrs	18	NR	NR	0.95	0.57	-
MMT	Vles, Kroes & Feron (2004) [26]	The Netherlands, Dutch	Performance measure	5–6 yrs	70	0.43–0.93	0.83–0.97	NR	NR	-
BOTMP	Bruininks & Bruininks (2005) [27]	US, English	Performance measure	4–21 yrs	Complete form: 53; Short form: 14	0.79	0.98	0.95–0.96	rho = 0.80	-
KTK	(Kiphard & Schilling, 2007) [28]	Germany, German	Performance measure	5–14 yrs	4	0.85	> 0.85	NR	NR	-
M-ABC 2 Motor test	Henderson, Sudgen & Barnett (2007) [29]	UK, English	Performance measure	3–17 yrs	3–6 yrs: 8; 7–10 yrs: 8; 11–16 yrs: 8	ICC: 0.94 ^a^	0.58 ^a^	0.70–0.76 ^a^	NR	Chinese, Dutch, Danish, Swedish, Italian, Japanese
M-ABC 2 Checklist	Henderson, Sudgen & Barnett (2007) [29]	UK, English	Self-report questionnaire	5–12 yrs	30	NR	NR	0.94 ^b^	0.35–0.44 ^c^	Chinese, Dutch, Danish, Swedish, Italian, Japanese
DCDQ ‘07	Wilson et al. (2009) [30]	Canada, English	Self-report questionnaire	5–15 yrs	15	0.94–0.97 ^d^	NR	0.89	0.76	Polish, Taiwanese, Brazilian, Italian, Dutch, French Canadian, German, Japanese, Spanish
APCM-2	Sabbadini (2015) [31]	Italy, Italian	Performance measure	2-8 yrs	2–3 yrs short form: 37; 2–3 yrs: 58; 37–48 mon: 77; 49–60 mon: 80; 61–72 mon: 76; 6.1–8 yrs: 56	NR	NR	>0.75	NR	-
EYMSC	Chambers & Sudgen (2006) [32]	UK, English	Self-report questionnaire	3–5 yrs	23	0.95	0.96	NR	−0.55	Norwegian, Portuguese, French, Dutch, German, Hebrew
ZNA	Kakebeeke et al. (2018) [33]	Switzerland, German	Performance measure	3–18 yrs	11	0.84	0.92	NR	NR	-

Legend: ICC, Interclass Correlation Coefficient; APCM–2, Abilità Prassiche e della Coordinazione Motoria—2nd Edition; BOTMP, The Bruininks–Oseretsky Test of Motor Proficiency; DCDQ ’07, Developmental Coordination Disorder Questionnaire; EYMSC, The Early Years Movement Skills Checklist; KTK, The Körperkoordinationtest für Kinder; M-ABC 2, The Movement Assessment Battery for Children 2; MAND, McCarron Assessment of Neuromuscular Development; MMT, Maastrichtse Motoriek Test; MOQ-T, The Motor Observation Questionnaire; MOT 4–6, The Motoriktest für vier- bis sechsjährige Kinder; NR, not reported; PDMS-2, The Peabody Developmental Motor Scales—2nd Edition; TGMD-2, The Test of Gross Motor Development–2nd Edition; ZNA, The Zurich Neuromotor Assessment. ^a^ From the validation study by Smits-Engelsman et al. [34] on a Dutch sample. ^b^ From validation study by Schoemaker et al. [35] on a Dutch sample. ^c^ From validation study by Schoemaker et al. [25] on a Dutch sample.^d^ From the Chinese [36] and Brazilian [37] versions of the test.
